# Preventing the return of fear using reconsolidation updating and methylene blue is differentially dependent on extinction learning

**DOI:** 10.1038/srep46071

**Published:** 2017-04-11

**Authors:** Allison M. Auchter, Jason Shumake, Francisco Gonzalez-Lima, Marie H. Monfils

**Affiliations:** 1 The University of Texas at Austin, Department of Psychology, Austin, TX 78712, USA; 2 Institute for Mental Health Research, The University of Texas at Austin, USA

## Abstract

Many factors account for how well individuals extinguish conditioned fears, such as genetic variability, learning capacity and conditions under which extinction training is administered. We predicted that memory-based interventions would be more effective to reduce the reinstatement of fear in subjects genetically predisposed to display more extinction learning. We tested this hypothesis in rats genetically selected for differences in fear extinction using two strategies: (1) attenuation of fear memory using post-retrieval extinction training, and (2) pharmacological enhancement of the extinction memory after extinction training by low-dose USP methylene blue (MB). Subjects selectively bred for divergent extinction phenotypes were fear conditioned to a tone stimulus and administered either standard extinction training or retrieval + extinction. Following extinction, subjects received injections of saline or MB. Both reconsolidation updating and MB administration showed beneficial effects in preventing fear reinstatement, but differed in the groups they targeted. Reconsolidation updating showed an overall effect in reducing fear reinstatement, whereas pharmacological memory enhancement using MB was an effective strategy, but only for individuals who were responsive to extinction.

Fear and anxiety-related disorders are the most prevalent psychiatric disorders. Accordingly, strategies for attenuating persistent fear have become important topics of study. Historically, the most widely used behavioral intervention for reducing fear responses has been extinction (clinically referred to as exposure therapy), whereby a subject is repeatedly exposed to a fearful stimulus in a safe environment, such that the emotional reaction to the stimulus diminishes. However, extinction is a complex process, and its long-term efficacy for reduction of fear is influenced by innumerable factors, including genetic variability (see ref. 1 for review), strength of the fear memory^2^, length of time since extinction^3^, parameters of extinction^4^,^5^ and a plethora of contextual stimuli^6^,^7^,^8^,^9^,^10^. In the present study, we examined the effectiveness of two memory-based paradigms in persistently reducing fear as a function of genetic variability in responsiveness to extinction (1) fear retrieval + extinction to attenuate the fear memory, and (2) pharmacological enhancement of extinction memory with low-dose USP methylene blue (MB). Using a 2 × 2 design, we tested each paradigm independently, as well as synergistically paired, in a population of rats that were selectively bred for their differential responding to extinction (high extinguishers, low extinguishers, and randomly bred^11^. The experiments assessed how genetic variability for fear extinction learning, reconsolidation updating of fear memory, responsiveness to extinction and pharmacological enhancement of extinction memory contribute to resilient fear reduction.

## Fear extinction

In non-human animal research, fear acquisition is typically modeled using classical (i.e. Pavlovian) conditioning^12^,^13^,^14^,^15^. In rodent fear conditioning, a previously neutral stimulus (conditioned stimulus; usually a tone or light) is paired with an aversive stimulus (unconditioned stimulus; usually a mild electric footshock). During extinction, the conditioned stimulus (CS) is presented repeatedly in absence of the unconditioned stimulus (US). This leads to a state in which the CS is no longer predictive of an aversive event, thus the conditioned response (CR; freezing) declines^16^,^17^,^18^,^19^,^20^. While this strategy for reduction of fear has shown some clinical efficacy, current behavioral models suggest that extinction alone does not result in lasting changes to the original blueprint of the fear memory itself^8^,^21^,^22^,^23^,^24^,^25^. Instead, extinction triggers the formation of a new inhibitory memory^8^,^22^,^26^,^27^,^28^,^29^,^30^. This results in two memories that compete for expression upon future CS-evoked memory retrieval^23^,^30^,^31^. If upon memory retrieval the memory for extinction is stronger than the original fear memory, then the extinction memory inhibits behavioral fear responses, and the animal does not respond in a fearful manner. If upon retrieval the extinction memory is not strong enough to suppress the fear responses (e.g. due to weak extinction or strong re-emergence of the original fear), then the animal will respond fearfully (e.g. freeze). This phenomenon leaves the subject susceptible to fear return due to the passage of time (spontaneous recovery^30^), return to the fearful context (renewal^21^) or re-exposure to the US (reinstatement^16^,^17^,^18^,^32^,^33^,^34^). The subject would be less vulnerable to fear return if, upon memory retrieval, the original fear memory was altered, or if the extinction memory was made stronger than the fear memory.

### Reconsolidation memory updating

Reconsolidation refers to the process whereby retrieval of a memory after the initial consolidation has been found to render it labile, and susceptible to modification^35^,^36^,^37^. Recently, Monfils *et al*.^38^ devised a behavioral paradigm that capitalized on the strengths of extinction and reconsolidation principles. In this paradigm, extinction training is presented after an isolated fear retrieval trial. This paradigm was found to result in a persistent reduction in fear expression, possibly suggesting that the original fear memory had been updated. This paradigm has been replicated several times in both non-human animals^39^,^40^,^41^,^42^,^43^,^44^,^45^,^46^,^47^,^48^ and humans^49^,^50^,^51^,^52^,^53^,^54^,^55^. However, there is a large degree of individual variability in extinction alone, and equally as much (if not more) variation in the retrieval + extinction paradigm. Furthermore, there have been a number of instances in which the post-retrieval extinction manipulation did not prevent the return of fear^48^,^52^,^56^,^57^. Thus, it is important not only to describe the optimal parameters for the retrieval + extinction paradigm, but also to investigate the implications of individual variability, since they may be important boundary conditions to the efficacy of the post-retrieval manipulation.

### Individual differences in fear extinction: the role of genetic variability

Though often overlooked, there is considerable individual variation in responsiveness to extinction, and genetic factors represent a significant source of this variation^1^. Indeed, there are significant individual differences in susceptibility to conditioning^58^ and extinction^59^ in both humans^60^,^61^,^62^ and other animals^11^,^63^,^64^. Additionally, anxiety disorders show strong heritability^65^, with approximately one-third of the variance in both human fear conditioning^1^,^58^ and anxiety disorders^66^ attributed to genetic factors.

Such factors may also play a role in memory processing during extinction, and should be considered when evaluating extinction’s effectiveness. However, the large number of potential genetic variants that could influence fear and extinction makes a comprehensive genetic assessment difficult. Instead of investigating the role of specific gene variants, we assessed extinction memory interactions using rats selectively bred for fear extinction behavior: high extinguishers (a lineage particularly responsive to extinction), low extinguishers (a lineage particularly unresponsive to extinction), and a randomly bred lineage^11^.

### Memory enhancement via metabolic enhancer methylene blue

Most pharmacological treatments for fear and anxiety-related disorders alter the action of specific neurotransmitter systems, acting directly on pathways involved in fear acquisition and maintenance; yet, recent work proposes that targeting memory and enhancing cognition may represent an alternative approach to the reduction of fear and anxiety symptoms^67^,^68^. One potential strategy is to use pharmacological agents that do not directly alter neurotransmission, but instead target neuronal mitochondrial respiration to boost the energy metabolism of a neuronal network, rather than a specific neurotransmitter/receptor system.

Methylene blue (MB) is a historical blue dye with the ability to do just that. Originally developed as a dye for the textile industry in 1876 by Heinrich Caro^69^, MB was applied by Ehrlich and Guttman^70^ to the treatment of malaria starting in 1891, and has been described as the first fully synthetic drug used in medicine^71^,^72^,^73^. Its medical use has since been greatly extended (see ref. 73 for a review), and it remains on the World Health Organization’s List of Essential Medicines as one of the most important medications needed in a basic health system^74^.

Diverging from typical pharmacological treatments, MB’s unique molecular structure allows it to cross the blood-brain barrier and accumulate in nervous tissue, particularly in mitochondria^75^. There, MB at low doses forms a redox equilibrium with electron transport chain complexes, resulting in increased oxygen consumption and ATP production, as well as decreased formation of damaging reactive oxygen species^76^,^77^,^78^,^79^,^80^,^81^,^82^. MB’s ability to enhance mitochondrial respiration has been demonstrated as a unique strategy for memory enhancement^78^,^83^,^84^,^85^, including enhancement of memory for extinguished fear^86^,^87^. MB’s action in these cases is to enhance mitochondrial respiration after fear extinction learning, thus enhancing extinction memory retention. One caveat to this approach is the fact that MB enhances any neural networks active during the time period MB is on board, regardless of whether the activation of those networks is beneficial. This means that administering MB after a poor learning experience may be detrimental because MB could theoretically strengthen networks that are interfering with learning^88^. Effectively, important studies have previously shown that pharmacologically enhancing extinction using D-Cycloserine is only effective when DCS is administered after an effective extinction session, suggesting that within-session fear reduction is important to an effective outcome^89^,^90^,^91^. The present experiment is the first investigation in rats of how MB’s therapeutic effectiveness may also depend on what is or is not learned during the task. We hypothesized that MB administration would be therapeutically beneficial to extinction learning for only those subjects who respond favorably to extinction training. Conversely, we predicted that MB would not be therapeutic—or even detrimental—to those subjects who do not respond to extinction training.

### Combining fear extinction, genetic variability, reconsolidation updating and memory enhancement in a model of persistent fear reduction

Fear reduction via extinction is a complex process that can be influenced by many factors. In this experiment, we tested several of these factors—genetic variability, reconsolidation updating and memory enhancement via MB—in combination in order to clarify which factors are the most important for persistent fear reduction. We hypothesized that subjects with differential extinction behavior would show differential responses to both the retrieval + extinction manipulation and post-extinction treatment with MB. Specifically, subjects who responded optimally to extinction (i.e., demonstrated large decreases in freezing during extinction) would benefit the most from both the memory updating paradigm and MB treatment. Those subjects who did not respond well to extinction (i.e., demonstrated no decreases or small decreases in freezing during extinction) would not benefit from the memory updating manipulation or MB treatment.

## Methods and Materials

### Subjects

Procedures were conducted in compliance with the National Institutes of Health Guide for the Care and Use of Experimental Animals and were approved by the Animal Care and Use Committee of the University of Texas at Austin.

### Selective breeding—parent generations

Subjects were selected from the third and fourth generations of a population of Long-Evans rats bred specifically for extinction phenotype (see ref. 11 for a detailed description of selection criteria and breeding procedures). Briefly, for four generations, Long-Evans rats were fear conditioned to a pure tone (20 s, 5 kHz, 80 dB) co-terminating with a footshock (0.7 mA for 0.5 s). The following day, subjects received 18 extinction trials (CS only). On the third day, rats received 3 memory-recall trials (CS only).

Founding breeders for the low extinguisher (LE) and high extinguisher (HE) lines were chosen by a two-step procedure that selected for differences in extinction in the absence of differences in acquisition^11^. A randomly bred (RB) control line was started from a random selection of males and females from the sample that were not chosen for the LE or HE lines.

### Husbandry

Throughout all breeding and experimental procedures, subjects were housed in temperature and humidity-controlled transparent polyethylene cages and were maintained on a 12 h/12 h light/dark cycle with food and water available ad libitum. Breeding pairs were housed together (1 male to 1 female) for 10 days (i.e., the average length of 2 estrous cycles). Females were then re-housed with their former female cage mate for the next 10 days, and then single housed until giving birth. On the day after birth, newborns were briefly separated from their mothers for sexing and culling. The number of males and females were counted, and litters were reduced in size to 12 pups (ideally to 6 males and 6 females or to the most equal sex ratio possible). The litters were then left undisturbed except for weekly cage changes until weaning at 21 days. From weaning until 41 days, rats were group housed with their same-sex siblings, usually in groups of 6. Thereafter, rats were housed in pairs.

### Experimental subjects

Subjects initially consisted of 96 male rats (approximately 60 days of age) selected from the third and fourth generations of the RB (n = 43), LE (n = 25), and HE (n = 28) lines described above. Within each line, rats were randomly assigned to each cell of a 2 × 2 (train × drug) factorial design: 1) extinction followed by saline, 2) retrieval + extinction followed by saline, 3) extinction followed by MB, or 4) retrieval + extinction followed by MB. Unfortunately, 3 video files became corrupted, leading to the loss of extinction-session data for 3 LE subjects, all of which had been assigned to the extinction + saline condition. In addition, after analyzing the effect of lineage on acquisition learning on the remaining 93 rats, we excluded an additional 9 subjects that failed to demonstrate conditioned fear (3 RB rats from the extinction + saline condition, 1 LE rat and 1 RB rat from the retrieval + extinction + saline condition, 2 RB rats from the extinction + MB condition, and 1 HE rat and 1 RB rat from the retrieval + extinction + MB condition) before analyzing the effect of lineage on extinction and the effects of retrieval + extinction and MB on reinstatement. Thus, the final sample size for the 2 × 2 factorial design consisted of 84 rats: extinction + saline (n = 19), retrieval + extinction + saline (n = 19), extinction + MB (n = 24), retrieval + extinction + MB (n = 22).

### Apparatus

All experimental manipulations (fear conditioning, retrieval, extinction, reinstatement, reinstatement probe) were administered in the same context (operant conditioning chambers; Coulbourn Instruments, Whitehall, PA). Each chamber was equipped with a stainless-steel rod flooring connected to a shock generator (Model H10-11R-TC-SF; Coulbourn Instruments, Whitehall, PA) and individually enclosed in a sound-insulated box (Isolation Cubicle, Model H10-24T; Coulbourn Instruments, Whitehall, PA). Chambers were illuminated with a red light. Behavior was recorded by infrared digital cameras (Panasonic, model wvBP334, Osaka, Japan) mounted on the ceiling of each unit. Stimulus presentation was automated using FreezeFrame2 software (Coulbourn Instruments, Whitehall, PA). Equipment was cleaned with Windex (SC Johnson, Racine, WI) between each session.

### Experimental procedures

#### Habituation to the context

To minimize contextual fear conditioning, on the day before fear conditioning, subjects were placed in the conditioning chambers for 15 minutes and allowed to freely explore. Neither the CS nor the US was given during this session.

#### Fear conditioning

**Figure 1:**
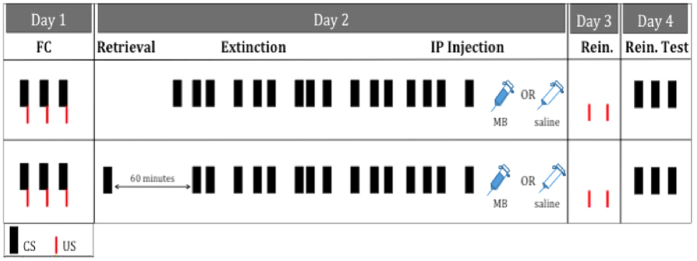
Experimental timeline. On Day 1, subjects received three tone-shock pairings. On Day 2, subjects received either a retrieval CS followed by extinction or extinction alone. Immediately following extinction, subjects received intraperitoneal injections of either saline or MB. On Day 3 subjects were presented with two unpaired USs, followed by three unpaired CSs the following day to test for reinstatement. FC = fear conditioning, Rein = reinstatement, CS = conditioned stimulus, US = unconditioned stimulus, MB = methylene blue, USP, 4 mg/kg. The bars, indicative of CS presentations, are drawn proportionally to inter-CS intervals.

A pictorial representation of the experimental timeline is illustrated in Fig. 1. Subjects were placed in the conditioning chambers, allowed to habituate for 3 minutes, and then fear conditioned with 3, 20-s, 5 kHz, 80 dB tones (CS). Each co-terminated with a 0.5-s, 0.7 mA footshock (US). The interval between each CS was 120 s. After conditioning, each subject remained in the chamber for 3 minutes and was then returned to its home cage.

#### Retrieval and extinction

The day after conditioning, subjects were returned to the conditioning chambers and allowed to habituate for 3 minutes. Following habituation subjects received a retrieval (CS) trial. After retrieval, subjects either remained in the chambers and received 18 more CSs (extinction alone group) or were placed back in their home cages, then returned to the conditioning chambers 60 min later to complete the 18-CS extinction (retrieval + extinction group). The interval between CSs was variable, ranging from 20 sec to 240 sec with an average of 120 sec. Upon completion of extinction, subjects remained in the conditioning chambers for 3 minutes. All subjects spent the same total amount of time in the context and were exposed to the same number of CSs (19 total). Subjects in the retrieval + extinction group spent the 60-minute duration between the retrieval CS and extinction with their cage-mates in the colony room.

#### Drug administration

MB was dissolved in sterile saline to a concentration of 5 mg/ml. Prior to returning to their home cages after extinction, subjects received an intraperitoneal (IP) injection of either 4 mg/kg USP MB or an equivalent volume of sterile saline.

#### Reinstatement and reinstatement test

The day after extinction, subjects habituated to the fear conditioning chambers for 5 minutes and then received two footshocks (0.5 s, 0.7 mA). The interval between footshocks was 120 s. Following the shocks, subjects remained in the chambers for 5 minutes. The following day, rats were placed back into the chambers and habituated for 5 minutes. To test for reinstatement of the tone-shock fear association, subjects heard 3 presentations of the CS alone.

### Behavioral scoring: freezing and residualized change

Freezing was defined as the absence of all movement aside from breathing and ear twitching, not including sleeping or resting. Behavior was scored manually from videos by an experimenter blind to experimental conditions. The total amount of CS-induced freezing was expressed as a percentage of total time spent freezing during each 20-s CS. Our initial plan was to operationalize post-acquisition conditioned fear as mean freezing over the first 3 tone-alone trials 24 hours after acquisition (i.e., the first 3 trials of extinction), post-extinction conditioned fear as mean freezing over the last 3 trials of extinction, and post-reinstatement conditioned fear as mean freezing over three CS presentations 24 hours after reinstatement. This plan was contingent on the statistical demonstration that variance between trials for these 3-trial blocks was random with respect to all independent variables and therefore ignorable. As detailed at the beginning of the Results, this turned out to be true only for the 3 trials at the end of extinction. Consequently, this was the only block of trials that we averaged over, and we limited analysis of acquisition and reinstatement behavior to the first CS-alone presentation following each of these sessions.

In addition, we operationalized within-session extinction and reinstatement as residualized change in freezing between initial post-acquisition freezing and these respective time points. Residualized change is a difference score defined by *Y* – *Y’*, where *Y’* is the regression of a subsequent freezing score, *Y*, on the initial freezing score, *X*. This partials out the variance attributed to individual differences in acquisition learning, rendering the residualized change scores linearly independent from (uncorrelated with) initial conditioned freezing levels. While this could also be accomplished by including initial freezing as a covariate in models that predict subsequent freezing behavior, regressing out acquisition variability prior to running subsequent models has the advantage of simplifying model output and interpretation and enabling us to plot and visualize extinction and reinstatement scores that are “uncontaminated” by differences in acquisition. Thus, these scores offer an *individualized, relativistic* measure of change, e.g., the residualized extinction score reflects how well an individual rat extinguishes relative to how well it is expected to extinguish given its initial level of fear.

### Statistical analyses

Data were analyzed and visualized using the R environment (version 3.3.2) for statistical computing and graphics, using a combination of ordinary least squares (OLS) linear regression^92^ and restricted maximum likelihood (REML) linear mixed effects regression^93^ as described in the Results. Analysis of variance (ANOVA) tables with F statistics and P values were then computed for model fits by first fitting the full model and then removing single effects and comparing the reduced model to the full model. For mixed effect models fit with the “lme4” package, p-values were calculated with the “afex” package using the Kenward-Roger approximation for degrees-of-freedom^94^.

The sequence of analyses followed the sequence in which the different experimental variables were introduced. First, preliminary regression models were used to evaluate the validity of averaging across trials and to generate residualized scores for extinction and reinstatement that control for differences in acquisition (see “Behavioral scoring” above). Second, lineage was a preexisting condition, so initial freezing was regressed on lineage. Third, the retrieval manipulation was introduced prior to extinction, so extinction scores were regressed on lineage and retrieval + extinction factors. Finally, the MB manipulation was introduced after extinction, so reinstatement scores were regressed on the factors of MB and retrieval + extinction. Lineage was not included in this last model; instead, we used observed extinction behavior (of which lineage is an imperfect predictor) as a continuous covariate to assess whether the effects of MB and/or retrieval + extinction are conditional on successful extinction learning.

## Results

### Retrieval + Extinction effects were trial dependent

As described in the “Behavioral scoring” section of the Methods, we planned to use the mean freezing over the first 3 CSs of the extinction session as an indicator of acquisition, the mean freezing over the last 3 CSs of the extinction session as an indicator of extinction, and the mean freezing over all 3 post-reinstatement CSs as an indicator of reinstatement. This reduction of 9 measurements into 3 blocks would presume that the independent variables only predict variation between blocks, not within blocks, such that within-block variation can be ignored. However, there is reason to question this assumption, especially for this experimental design. For example, the HE line might produce subjects that undergo such rapid extinction that there would be differential freezing by the third CS-alone trial even if there is no difference to the first CS-alone trial, or the delay between the first and second CS-alone trials for the group that receives an extra retrieval trial prior to extinction might cause a differential response to the second trial. We attempted to rule out the presence of such trial interactions by fitting a limited linear mixed effects model to each block of 3 trials, with a random effect of subject and fixed effects for each independent variable and its interaction with trial sequence. The goal of these models was not to test our specific hypotheses about the effects of the experimental variables on extinction learning and fear reinstatement, but rather to look for evidence that these variables interact with trial sequence. If we cannot rule out the presence of such interactions, then it would be better to use the first trial of each session as an indicator of long-term memory rather than averaging across trials.

### First three trials of extinction

**Figure 2:**
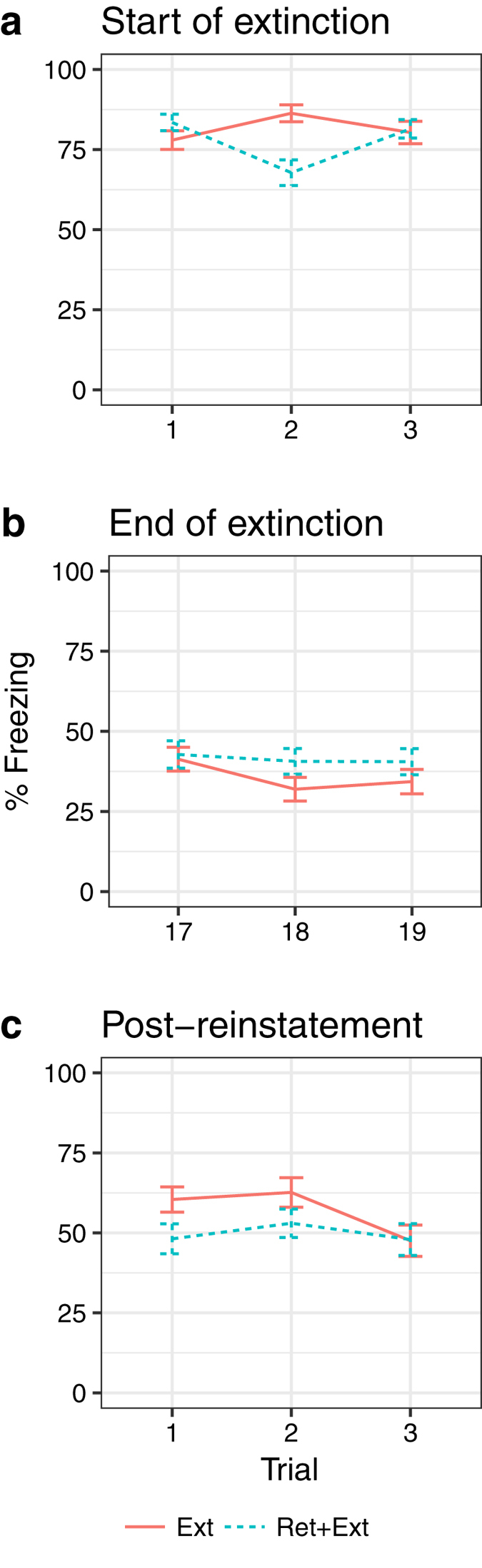
Freezing at (**a**) beginning of extinction, (**b**) end of extinction, and (**c**) post-reinstatement for the extinction vs. retrieval + extinction groups. A. Mean (SEM) showing that the extinction and retrieval + extinction groups did not differ in their freezing to the first trial of extinction, but did differ in their response to the second trial. There was a large interaction effect between trial and training type, F(2,178) = 12.0, p < 0.0001. B. There were no differences between the extinction and retrieval + extinction groups between the last 3 trials at the end of extinction. C. There was an interaction between trial and retrieval + extinction, F(2,176) = 3.0, p = 0.05, for fear reinstatement, with the effect strongest for the first trial and undetectable by the third trial.

At this point in our analysis, the MB manipulation had not yet been introduced, so we limited this model to the effects of lineage and retrieval + extinction. The mixed effects regression revealed strong evidence that the retrieval + extinction manipulation did in fact result in a differential response to the second trial (Fig. 2a). There was a large interaction effect between trial number and training type, F(2,178) = 12.0, p < 0.0001 (Supplementary Table 1a). Inspection of model coefficients (Supplementary Table 1b) confirmed that retrieval + extinction resulted in a differential quadratic trend over the first 3 trials. Based on this analysis, we determined it would not be advisable to average across the first three trials, as this would underestimate the fear acquisition of the retrieval + extinction group and introduce bias when evaluating extinction and reinstatement effects that co-vary for acquisition performance. For this reason, we used only the first trial of the extinction session to gauge fear acquisition in the subsequent analyses.

#### Last three trials of extinction

None of the experimental effects on end-of-extinction freezing were conditional on trial sequence, i.e., there were no interactions (Supplementary Table 2a, Supplementary Table 2b, Fig. 2b). Therefore, averaging across this block of trials would not mask any experimental effects.

#### Reinstatement

For this model, we added the effect of MB, which was introduced after the end of extinction. There was evidence that an effect of the retrieval + extinction manipulation on fear reinstatement (which was *a priori* hypothesized) was detectable during the first trial of the reinstatement test, but was not detectable by the third tone presentation (Fig. 2c). There was an interaction between trial number and retrieval + extinction, F(2, 176) = 3.0, p = 0.05 (Supplementary Table 3a). Inspection of model coefficients showed that retrieval + extinction resulted in a differential linear trend over trials (Supplementary Table 3b). Figure 2c shows that subjects that did not receive an isolated retrieval trial before extinction began to re-extinguish by the third trial. Note also in Supplementary Tables 3a and 3b that there was a significant effect of lineage on reinstatement freezing that was not dependent on trial, F(2, 88) = 5.09, p = 0.008, with the LE line freezing 13.8 (SE = 6.9) percentage points more than the RB line and the HE line freezing 10.1 (SE = 6.4) percentage points less. However, given that we cannot ignore the trial dependency of the retrieval + extinction effect, we only used the first trial to gauge fear reinstatement for all experimental effects.

### Lineage did not predict conditioned freezing after acquisition

**Figure 3:**
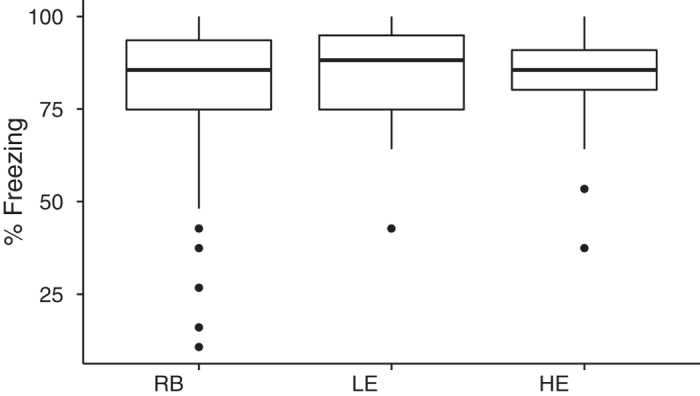
Lineage does not predict conditioned freezing after acquisition. Boxes represent the middle 50% of the distribution (the interquartile range between the 25th and 75th percentiles), and the horizontal line indicates the median. The “whiskers” that extend vertically from the box indicate the range of observations that fall within ±1.5 times the interquartile range, and any observations outside the whiskers are graphed as individual points. Individual differences in the acquisition of the CS-US association were not predicted by lineage, F(2,90) = 1.0, p = 0.35. Note, however, the variance for the randomly bred group was greater than the variance for the selected lines, with a much wider range of freezing behavior. RB = Randomly Bred (n = 43), LE = Low Extinguisher (n = 22), HE = High Extinguisher (n = 28).

Individual differences in the acquisition of the CS-US association were not predicted by lineage, F(2,90) = 1.0, p = 0.35. Note, however, in the boxplots of Fig. 3 that the variance for the randomly bred group was greater than the variance for the selected lines, with a much wider range of freezing behavior. This was to be expected, since the HE and LE lines were selected for convergent (high) acquisition and divergent extinction. As such, they should (and do) show less variance in acquisition than a randomly bred population. Having established that there were no line-dependent differences in acquisition, we excluded 9 subjects that demonstrated inadequate acquisition (<50% freezing) for the subsequent analyses of extinction learning and fear reinstatement. (The experimental conditions to which each of these subjects belonged is given in the Methods).

### Residualized change scores control for individual differences in acquisition

As described in the “Behavioral scoring” section of the Methods, we generated residualized change scores by regressing post-extinction freezing and post-reinstatement freezing on post-acquisition freezing. Very little (~5%) of the variance in post-extinction freezing was explained by individual differences in acquisition and virtually none of the variance in post-reinstatement freezing was explained. Nonetheless, to absolutely rule out any possible influence of acquisition differences on subsequent freezing measures, we used the residuals from these regressions (the variance in post-extinction freezing not explained by the variance in pre-extinction freezing) as indicators of within-session extinction learning and reinstatement in the subsequent analyses. By mathematical definition, residual scores are centered at zero. To generate extinction scores, we multiplied the residuals by −1 so that more positive values would reflect greater extinction (less freezing than expected given acquisition level) and more negative values would reflect less extinction (more freezing than expected given acquisition level). This multiplication by −1 was not done for reinstatement scores since greater-than-expected freezing indicates greater reinstatement as is. We also scaled the scores to have a standard deviation of 1 to facilitate subsequent interpretation. To summarize, these are standardized scores of extinction and reinstatement behavior that control for nuisance differences in acquisition.

### Lineage predicted within-session extinction learning

**Figure 4:**
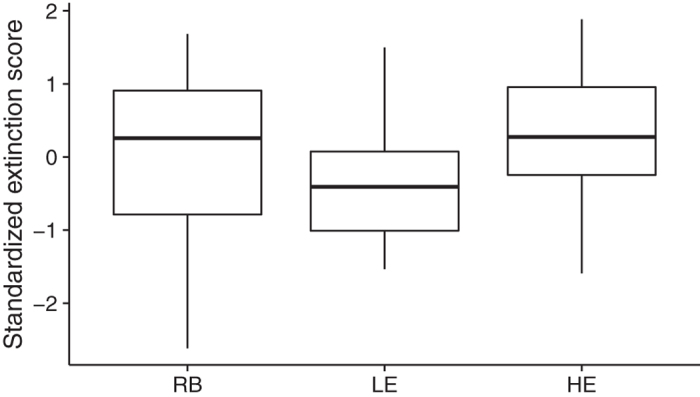
Lineage predicts within-session extinction learning. Boxplots show the standardized extinction scores for the RB, LE, and HE groups. Lineage was a non-significant predictor of extinction performance when including all 3 lines in the model, F(2,81) = 2.3, p = 0.11. However, it is evident from the boxplots that the variance was much greater for the RB group, which violates assumptions of homogeneity of variance. We refit the model to contrast only the selected lines, excluding RB rats. Doing so revealed that the contrast between the HE and LE lines was significant, F(1,46) = 6.1, p = 0.02, with heredity explaining 12% of the variance in within-session extinction learning. On average, HE rats showed extinction that was 0.6, 95% CI [0.11, 1.1], standard deviations better than LE rats. RB = Randomly Bred (n = 36), LE = Low Extinguisher (n = 21), HE = High Extinguisher (n = 27).

Lineage was a non-significant predictor of extinction performance when including all 3 lines in the model, F(2,81) = 2.3, p = 0.11. However, the boxplots in Fig. 4 show that one should be wary of including the RB group in a linear model evaluating line differences. This is because the variance in extinction performance was far greater in the RB group, which fully encompassed the distributions of both the LE and HE lines, a gross violation of the homogeneity of variance assumption that could lead to inaccurate standard errors and p values. This outcome is not surprising, given that the selectively bred lines were, by design, more homogeneous populations derived from a more heterogeneous randomly bred population. For this reason, we refit the model to contrast only the selected lines, excluding RB rats. Doing so revealed that the contrast between the HE and LE lines was significant, F(1,46) = 6.1, p = 0.02, with heredity explaining 12% of the variance in within-session extinction learning. On average, HE rats showed extinction that was 0.6, 95% CI [0.11, 1.1], standard deviations better than LE rats.

### Experimental manipulations targeting prevention of reinstatement

In the next set of analyses, we maximized power by pooling all lines together, and used observed within-session extinction learning as a continuous covariate along with MB and retrieval + extinction conditions to predict reinstatement. We first tested a model that included a 3-way interaction between achieved extinction learning, training condition (extinction vs. retrieval + extinction), and MB. There was no evidence of an extinction × training × MB interaction, F(1,76) = 0.01, p = 0.92, nor was there evidence of a training × MB interaction, F(1,77) = 0.37, p = 0.55. We next tested the 2-way interaction between extinction scores and MB, followed by the 2-way interaction between extinction scores and training condition.

### Effect of MB depended on successful extinction learning

**Figure 5:**
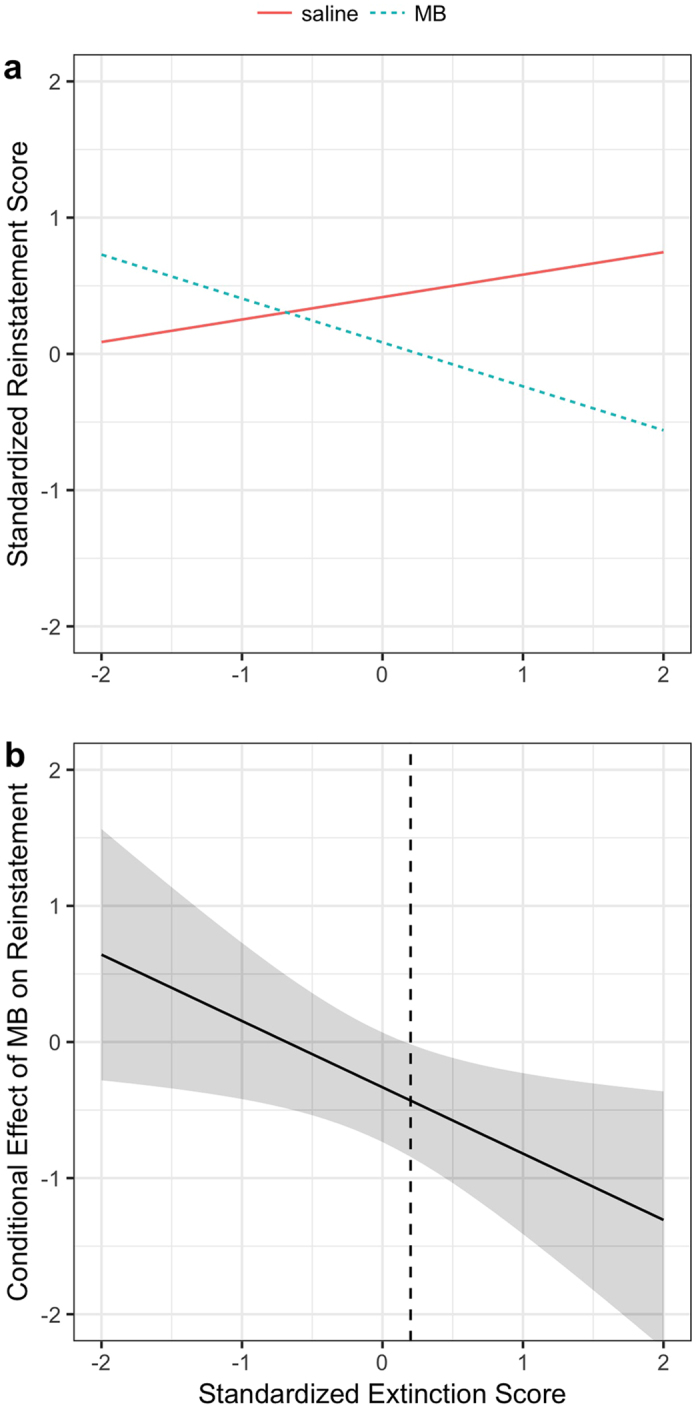
Effect of MB depended on successful extinction learning. (**a**) There was a tendency for the MB-treated group to show a greater reduction in fear reinstatement as a function of better within-session extinction learning while the opposite was true for the saline-treated group. (**b**) A conditional effect plot shows the projected *difference* between the MB and saline groups as a function of within-session extinction (the corresponding vertical distance between the two lines graphed above in panel a). The region where the 95% confidence interval of this difference does not include zero indicates the range of extinction performance where there is a significant difference between the treatment groups (p < 0.05). This analysis indicates that MB caused a significant reduction in reinstatement relative to saline controls if rats were at least 0.2 SDs above the sample mean extinction performance.

**Figure 6:**
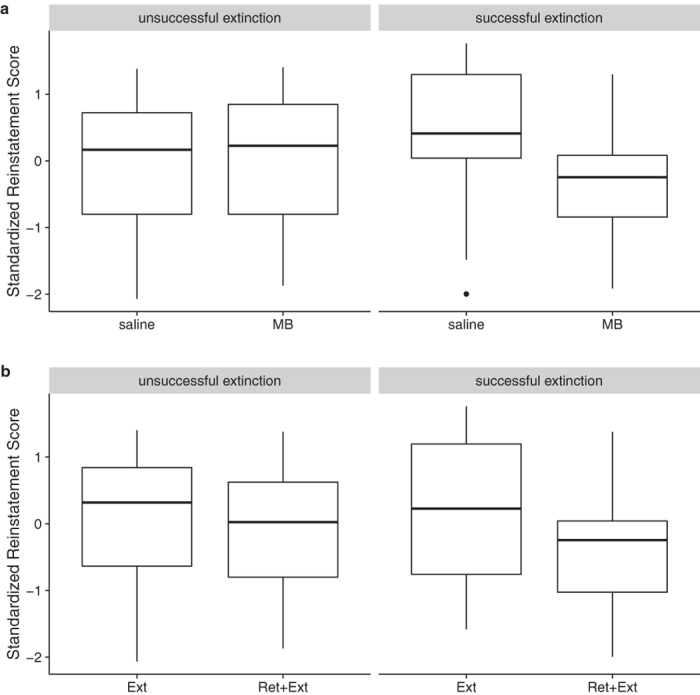
MB depends on successful extinction training; retrieval + extinction does not. (**a**) Boxplots for the MB vs. saline groups after splitting the sample into Unsuccessful vs. Successful extinction based on the 0.2 SD cutoff described in the results and Fig. 5b. Welch’s t-tests revealed that the difference between the MB and saline groups for the Unsuccessful (n = 46) extinction was not significant, t(41.5) = −0.34, p = 0.73, but the difference for the Successful (n = 38) extinction was, t(30.1) = 2.18, p = 0.03. (**b**) There was no evidence for an extinction × training interaction, F(1,78) = 0.29, p = 0.59. There was, however, a significant independent effect of retrieval + extinction, F(1,79) = 5.6, p = 0.02, which resulted in a reduction in fear reinstatement of 0.5 standard deviations, 95% CI [0.08, 0.92]. To compare this with the conditional effect of MB, we likewise created separate plots of the retrieval + extinction effect for Successful vs. Unsuccessful extinction groups. While the effect of retrieval + extinction appears more noticeable in the Successful extinction group in terms of the interquartile range, the difference between the medians is of a similar direction and magnitude for both Successful and Unsuccessful extinction groups.

There was marginal evidence for an extinction × MB interaction, F(1,79) = 2.9, p = 0.09. If we exclude the subject exerting the most influence on model fit statistics as given by Cook’s distance (a high-extinction subject that received saline and showed no reinstatement), the extinction × MB interaction appears stronger, F(1,78) = 5.1, p = 0.03. We therefore conducted follow-up tests to probe a potential moderation effect of extinction learning on the efficacy of MB. Figure 5a shows a tendency for the MB-treated group to show a greater reduction in fear reinstatement as a function of better within-session extinction learning while the opposite was true for the saline-treated group. To determine if there was a significant divergence between the functions shown in Fig. 5a, we constructed a conditional effect plot (Fig. 5b), which shows the projected difference between the MB and saline groups as a function of within-session extinction. The region where the 95% confidence interval of this difference does not include zero indicates the range of extinction performance where there is a significant difference between the treatment groups (p < 0.05). This analysis indicates that MB caused a significant reduction in reinstatement relative to saline controls if rats were at least 0.2 SDs above the sample mean extinction performance. This benchmark approximately translates to a 58% reduction in freezing from the beginning to the end of the extinction session. Figure 6a shows the plots for the MB vs. saline groups after splitting the sample into Unsuccessful vs. Successful extinction based on this cutoff. As revealed by Welch’s t-tests, the difference between the MB and saline groups for the Unsuccessful (n = 46) extinction was not significant, t(41.5) = −0.34, p = 0.73, but the difference for the Successful (n = 38) extinction was, t(30.1) = 2.18, p = 0.03. If the outlier, shown as an isolated point in the successful extinction saline group (Fig. 6a), is excluded, the difference for Successful extinction is more pronounced, t(30.9) = 2.9, p = 0.008.

### Effect of retrieval + extinction did not depend on successful extinction learning

There was no evidence for an extinction × training interaction, F(1,78) = 0.29, p = 0.59. There was, however, a significant independent effect of retrieval + extinction, F(1,79) = 5.6, p = 0.02, which resulted in a reduction in fear reinstatement of 0.5 standard deviations, 95% CI [0.08, 0.92]. To compare this with the conditional effect of MB, we likewise created separate plots of the retrieval + extinction effect for Successful vs. Unsuccessful extinction groups in Fig. 6b. While the effect of retrieval + extinction appears more noticeable in the Successful extinction group in terms of the interquartile range, the difference between the medians is of a similar direction and magnitude for both Successful and Unsuccessful extinction groups.

### Summary

Overall, the best model included a main effect of retrieval + extinction training that was independent of extinction learning, and an effect of MB that was dependent on extinction training. Including all subjects, this model explained 13.5% of the variance in reinstatement, F(4,79) = 3.08, p = 0.02. Excluding the previously mentioned outlier, this model explained 14.7% of the variance in reinstatement, F(4,78) = 3.37, p = 0.01.

## Discussion

This experiment examined the efficacy of two strategies in persistently reducing conditioned fear—reconsolidation memory updating and extinction memory enhancement with MB. We tested our predictions in subjects selectively bred for extinction phenotype and observed how their genetic predisposition influenced their actual extinction behavior and, in turn, how their actual extinction behavior modulated the effects of our two strategies.

### Selective breeding for extinction phenotype does not predict conditioned fear after acquisition, but does predict within-session extinction

Our results showed no differences between our three lines (RB, LE, HE) on freezing after acquisition. When examining whether lineage was predictive of within-session extinction learning, we found a marginal effect when all three lines were compared and a significant difference when strictly comparing the HE and LE lines. On average, the HE line extinguished better than the LE line. This was not surprising, in light of the fact that the different lines were specifically bred to show differences in extinction.

### Successful extinction learning moderates MB’s effect on reinstatement

Our results suggest that post-training MB was effective only in subjects that were responsive to behavioral extinction (the successful extinguishers). MB’s action as a neurometabolic enhancer does not discriminate between successful and unsuccessful extinction training. In other words, post-training MB administration is hypothesized to enhance the consolidation of whatever brain network was active during a task. Thus, if a subject remained fearful throughout extinction, then post-training treatment with MB should enhance the memory of that fear-evoking brain network. On the other hand, if fear was reduced during extinction, then post-training MB administration should be beneficial by enhancing the fear-reducing/fear reduced brain network. This hypothesis, that extinction learning would moderate the effect of MB, was supported by our results, which are consistent with recent findings of MB’s effect on extinction in humans with claustrophobia^88^. Stated broadly, MB could be utilized as an alternative to (or potentially in conjunction with, though this would require further investigation) traditional pharmacological treatments in cases of successful fear reduction by exposure therapy.

### Benefits of retrieval + extinction do not depend on successful extinction learning

Monfils *et al*.^38^ demonstrated that extinction presented during the reconsolidation window prevents reinstatement of fear. We replicated this finding by showing an overall main effect of extinction type (retrieval + extinction vs. extinction alone) on reinstatement freezing, with the retrieval + extinction condition freezing less than the extinction alone condition during the post reinstatement test. This effect was not moderated by extinction success, and it was additive (as opposed to synergistic) with the effect of MB. Post-retrieval extinction (or reconsolidation updating) offers a promising treatment avenue, since with a simple modification to exposure therapy, there is the potential for a more persistent memory modification; however, there is mounting evidence suggesting that it may be quite susceptible to boundary conditions^95^. The present results may suggest that extinction success does not act as a boundary condition.

### The bottom line

Our findings support two major points: (1) both MB and retrieval + extinction serve to persistently reduce conditioned fear, but (2) only MB’s action depends on the success of an extinction session. Thus, MB is effective at enhancing memory consolidation, particularly for extinction, but its use as a clinical treatment for fear and anxiety disorders should be limited to individuals who respond favorably to extinction (similarly to DCS). Our results further suggest that the ability to experience a successful extinction session—and thereby become a candidate for a memory-enhancing intervention like MB—is constrained by heredity. For example, only 5 out of 21 rats from the LE line met the criterion for a predicted benefit of MB. Perhaps with additional extinction sessions these rats would reach a point at which they too would benefit from MB, or perhaps genetically vulnerable populations that are resistant to extinction will require interventions that disrupt pathological memories directly or otherwise do not depend on extinction learning.

In light of MB’s effect depending on within-session extinction, it is also important to point out that, as was previously reported, within-session extinction is not necessarily a good predictor of between-session fear reduction^96^. If individuals do not extinguish conditioned fear responses, then post-training MB is likely to only enhance (re)consolidation of fear memories, as a memory for extinction formed weakly or perhaps not at all. Reconsolidation updating (using retrieval-extinction) provides a potentially useful avenue for treating anxiety-relates disorders, but more work needs to be conducted to establish the optimal parameters of administration and improve our understanding of boundary conditions that limit or prevent its efficacy. The present work is an important step in identifying which individual phenotypes might best benefit from available treatment avenues.

## Additional Information

**How to cite this article**: Auchter, A. M. *et al*. Preventing the return of fear using reconsolidation updating and methylene blue is differentially dependent on extinction learning. *Sci. Rep.* **7**, 46071; doi: 10.1038/srep46071 (2017).

**Publisher's note:** Springer Nature remains neutral with regard to jurisdictional claims in published maps and institutional affiliations.

## Supplementary Material

Supplementary Tables
