# Supplementary material for: Preventing the return of fear using reconsolidation updating and methylene blue is differentially dependent on extinction learning
Source: Sci Rep. 2017 Apr 11;7:46071. doi: 10.1038/srep46071 (PMC5387397; doi:10.1038/srep46071)
Supplement: Supplementary Tables [file srep46071-s1.docx]

**Preventing the return of fear using reconsolidation updating and methylene blue is differentially dependent on extinction learning**

Allison M. Auchter^1^, Jason Shumake^1,2^, Francisco Gonzalez-Lima, Marie H. Monfils*

The University of Texas at Austin, Department of Psychology, Austin, TX 78712, USA

**Supplementary materials**

^1^ These authors contributed equally

^2^ Institute for Mental Health Research, The University of Texas at Austin

*Corresponding Author: Marie H. Monfils

The University of Texas at Austin

Department of Psychology

108 E. Dean Keeton Stop A8000

Austin, TX 78712, USA

(512) 471-4139

marie.monfils@utexas.edu

Table 1. First 3 Trials of Extinction

Table 1A. ANOVA for full model

| Effect | Df | F | p |
| --- | --- | --- | --- |
| Trial | 2, 178 | 2.05 | .13 |
| Lineage | 2, 89 | 0.75 | .47 |
| Ret+Ext | 1, 89 | 1.61 | .21 |
| Trial × Lineage | 4, 178 | 0.96 | .43 |
| Trial × Ret+Ext | 2, 178 | 11.95 | <.0001 |

Table 1B. Coefficients for Trial * Ret+Ext model

|  | Estimate | Std. Error | t value |
| --- | --- | --- | --- |
| (Intercept) | 81.540 | 2.279 | 35.78 |
| Ret+Ext | -3.964 | 3.276 | -1.21 |
| Trial (linear) | 1.686 | 2.527 | 0.67 |
| Trial (quadratic) | -5.867 | 2.527 | -2.32 |
| Ret+Ext × Trial (linear) | -3.098 | 3.633 | -0.85 |
| Ret+Ext × Trial (quadratic) | 17.860 | 3.633 | 4.92 |

Table 2. Last 3 Trials of Extinction

Table 2A. ANOVA for full model

| Effect | Df | F | p |
| --- | --- | --- | --- |
| Trial | 2, 178 | 3.85 | .02 |
| Lineage | 2, 89 | 2.75 | .07 |
| Ret+Ext | 1, 89 | 1.13 | .29 |
| Trial × Lineage | 4, 178 | 1.31 | .27 |
| Trial × Ret+Ext | 2, 178 | 1.02 | .36 |

Table 2B. Coefficients for Trial + Lineage model

|  | Estimate | Std. Error | t value |
| --- | --- | --- | --- |
| (Intercept) | 37.587 | 3.309 | 11.360 |
| LE - RB | 9.950 | 5.687 | 1.750 |
| HE - RB | -4.801 | 5.269 | -0.911 |
| Trial (linear) | -3.322 | 1.922 | -1.728 |
| Trial (quadratic) | 2.871 | 1.922 | 1.493 |

Table 3. Post-Reinstatement Trials

3A. ANOVA for full model

| Effect | Df | F | p |
| --- | --- | --- | --- |
| Trial | 2, 176 | 2.51 | .08 |
| Lineage | 2, 88 | 5.09 | .008 |
| Ret+Ext | 1, 88 | 2.33 | .13 |
| MB | 1, 88 | 2.90 | .09 |
| Trial × Lineage | 4, 176 | 0.74 | .57 |
| Trial × Ret+Ext | 2, 176 | 2.96 | .05 |
| Trial × MB | 2, 176 | 0.03 | .97 |

3B. Coefficients for Line + MB + Trial × Ret+Ext model

|  | Estimate | Std. Error | t value |
| --- | --- | --- | --- |
| (Intercept) | 62.210 | 5.636 | 11.039 |
| LE – RB | 13.757 | 6.866 | 2.004 |
| HE - RB | -10.138 | 6.366 | -1.592 |
| MB | -9.307 | 5.463 | -1.704 |
| Ret+Ext | -8.305 | 5.435 | -1.528 |
| Trial (linear) | -9.115 | 2.698 | -3.378 |
| Trial (quadratic) | -7.063 | 2.698 | -2.618 |
| Trial × Ret+Ext (linear) | 8.955 | 3.879 | 2.309 |
| Trial × Ret+Ext (quadratic) | 2.983 | 3.879 | 0.769 |
